# Exploitation of algal-bacterial associations in a two-stage biohydrogen and biogas generation process

**DOI:** 10.1186/s13068-015-0243-x

**Published:** 2015-04-08

**Authors:** Roland Wirth, Gergely Lakatos, Gergely Maróti, Zoltán Bagi, János Minárovics, Katalin Nagy, Éva Kondorosi, Gábor Rákhely, Kornél L Kovács

**Affiliations:** Department of Biotechnology, University of Szeged, Közép fasor 52, H-6726 Szeged, Hungary; Institute of Biochemistry, Biological Research Center, Hungarian Academy of Sciences, Temesvári krt. 62, H-6726 Szeged, Hungary; Institute of Biophysics, Biological Research Center, Hungarian Academy of Sciences, Temesvári krt. 62, H-6726 Szeged, Hungary; Department of Oral Biology and Experimental Dental Research, University of Szeged, Tisza L. krt. 64, 6720 Szeged, Hungary

**Keywords:** Microalgae, Biogas, Biohydrogen, Algal bacterial co-culture, Metagenomics

## Abstract

**Background:**

The growing concern regarding the use of agricultural land for the production of biomass for food/feed or energy is dictating the search for alternative biomass sources. Photosynthetic microorganisms grown on marginal or deserted land present a promising alternative to the cultivation of energy plants and thereby may dampen the ‘food or fuel’ dispute. Microalgae offer diverse utilization routes.

**Results:**

A two-stage energetic utilization, using a natural mixed population of algae (*Chlamydomonas* sp. and *Scenedesmus* sp.) and mutualistic bacteria (primarily *Rhizobium* sp.), was tested for coupled biohydrogen and biogas production. The microalgal-bacterial biomass generated hydrogen without sulfur deprivation. Algal hydrogen production in the mixed population started earlier but lasted for a shorter period relative to the benchmark approach. The residual biomass after hydrogen production was used for biogas generation and was compared with the biogas production from maize silage. The gas evolved from the microbial biomass was enriched in methane, but the specific gas production was lower than that of maize silage. Sustainable biogas production from the microbial biomass proceeded without noticeable difficulties in continuously stirred fed-batch laboratory-size reactors for an extended period of time. Co-fermentation of the microbial biomass and maize silage improved the biogas production: The metagenomic results indicated that pronounced changes took place in the domain Bacteria, primarily due to the introduction of a considerable bacterial biomass into the system with the substrate; this effect was partially compensated in the case of co-fermentation. The bacteria living in syntrophy with the algae apparently persisted in the anaerobic reactor and predominated in the bacterial population. The Archaea community remained virtually unaffected by the changes in the substrate biomass composition.

**Conclusion:**

Through elimination of cost- and labor-demanding sulfur deprivation, sustainable biohydrogen production can be carried out by using microalgae and their mutualistic bacterial partners. The beneficial effect of the mutualistic mixed bacteria in O_2_ quenching is that the spent algal-bacterial biomass can be further exploited for biogas production. Anaerobic fermentation of the microbial biomass depends on the composition of the biogas-producing microbial community. Co-fermentation of the mixed microbial biomass with maize silage improved the biogas productivity.

## Introduction

Biomass utilization for energy generation is commonly regarded as a major contributor to the achievement of renewable energy production targets [1-4]. Energy carriers from biomass are currently predominantly produced through the use of terrestrial plants [5]. The intensive exploitation of land for the cultivation of crops destined for biofuel production, however, may exert a negative impact on the global supply and the price of food and feed [6].

The search for alternative biomass sources still continues. Economically and environmentally friendly solutions should be found. Huge energetic and biorefinery opportunities are offered by the conversion of solar energy via the use of photosynthetic microorganisms. Hence, the interest in photosynthetic microorganisms (and especially microalgae) is growing worldwide. The microalgae are a large and diverse group of microscopic, photoautotrophic, or photoheterotrophic organisms, which grow profusely in both salt and fresh natural waters [7]. Microalgae are able to double their biomass much faster than terrestrial plants, and they therefore produce more biomass per hectare than higher plants do [8]. The relatively small land area needed to cultivate microalgae may be arable or marginal land, which further decreases the competition for agricultural land and smothers the ‘food or fuel’ dispute [7]. Microalgae can be harvested practically all year round, hence improving the biomass production efficacy and eliminating numerous storage problems. Cultivation is possible in closed photobioreactors or in open ponds. Open systems are usually considered to be economical, while closed systems are more efficient from the aspect of biomass production and control of the cultivation parameters [9,10]; either concept may therefore be competitive in diverse applications [11]. Additional beneficial features of a microalgal biomass include versatility and the variety of utilization for energetic purposes such as biohydrogen (bioH_2_), bioethanol, biodiesel, and biogas production [12-14], besides biorefinery applications [14-16].

The important properties of a microalgal biomass to be used in anaerobic digestion (AD) include high contents of lipids and/or carbohydrates and a lack of recalcitrant lignin [12]. The lipid and carbohydrate content amounts up to 50% of the biomass dry weight in some strains [10,17]. Research on the AD of algal biomass started more than 50 years ago [18]. Until recently, only a few studies followed up this line of research [19-24]. Levels of biogas productivity from various fresh and salt water algal strains have been compared under mesophilic conditions [25]. The biogas potential was found to depend strongly on the species and on the cell disruption method applied. The CH_4_ content of the gas evolved from the microalgae was 7% to 13% higher than that from maize silage [25]. A closed-loop system to convert the algal biomass to biogas and electricity has been tested [26]. The microbial communities thriving in anaerobic digesters fed with algal biomass have not been investigated extensively. The archaeal community formed during microalgal fermentation was recently analyzed by next-generation sequencing [27].

Some microalgae, such as the most extensively studied green microalga *Chlamydomonas reinhardtii*, have the noteworthy ability to produce H_2_ via a photosynthetic water-splitting reaction coupled with the dark hydrolysis of storage materials [28-30]. Sulfur deprivation becomes a standard method through which to switch the algal metabolism from photoautotrophy to dark heterotrophic H_2_ generation. The two-step process during which the cells undergo major metabolic and biochemical changes demands considerable energy input both by the process operators and by the algae.

Naturally formed, mixed algal-bacterial microbial communities have been observed to have beneficial effects on algal growth [31-34]. The mutualistic relationship involves supplying the algae with important growth factors, notably vitamin B12, by the bacterial partner in exchange for organic nutrients [35-39]. Little is known about H_2_ production by algal-bacterial systems [40]. A recent study proposed that by consuming the O_2_ generated photosynthetically by the algae, the bacteria maintain an anaerobic environment suitable for algal bioH_2_ production [41]. This may eliminate the need for the sulfur-deprivation step [28-30].

In this study, we modeled a two-stage biorefinery process, that is, H_2_ production in the first stage by an algal-bacterial mixed biomass grown under nonsterile photoheterotrophic conditions, with biogas generation from the residual biomass in the second stage. The composition of the microalgal-bacterial mixture was monitored during the process by using next-generation DNA sequencing technology.

## Results and discussion

### H_2_ production by the mixed algal-bacterial system

**Figure 1:**
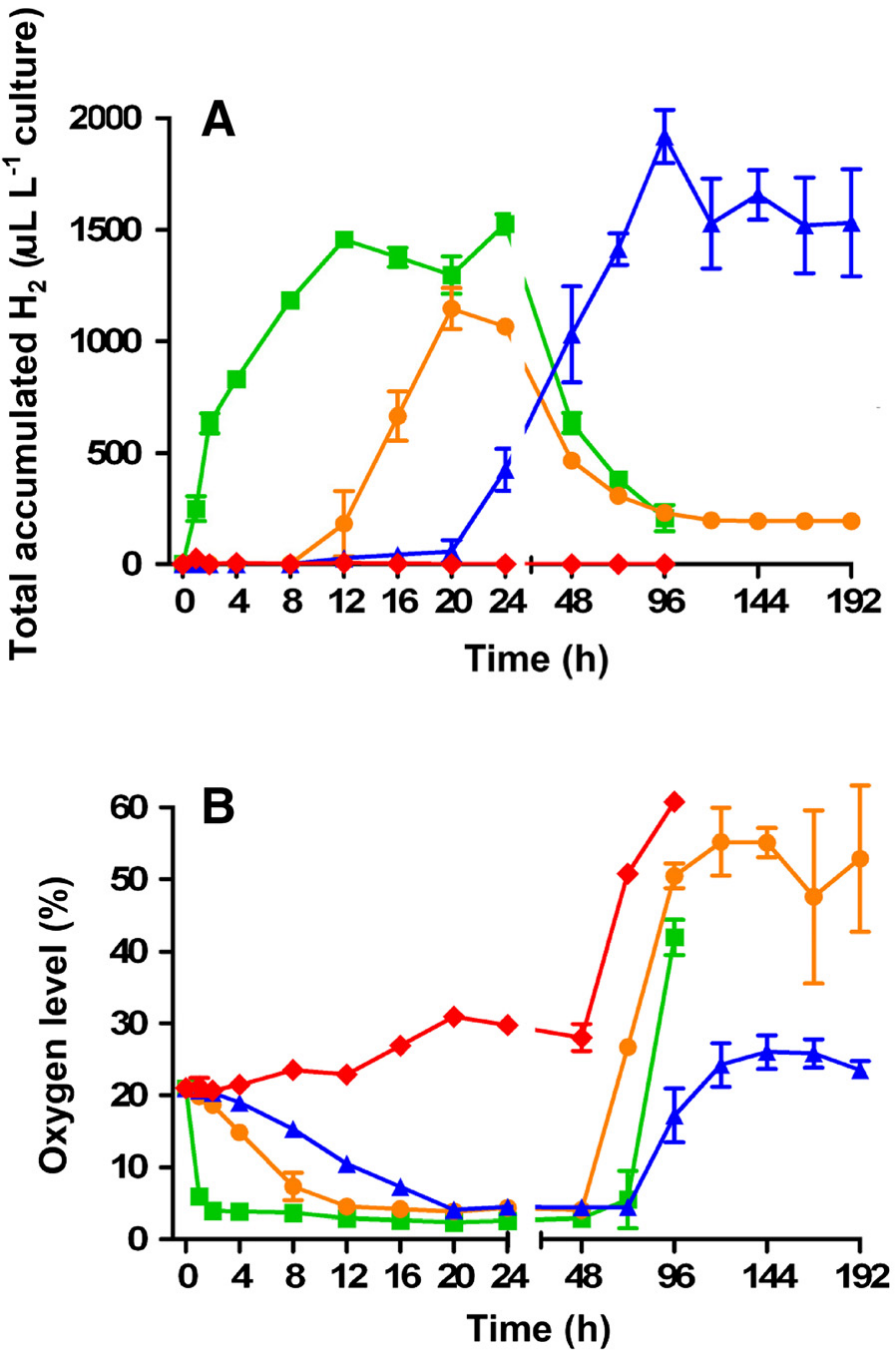
**H**_**2**_ **accumulation (A) and O**_**2**_ **content (B) in the headspaces of the various cultures in time.** Orange circles: mixed algal-bacterial co-culture (AB + S); green squares: algal-bacterial mixture with added *E. coli ΔhypF* (AE + S); blue triangles: sulfur-deprived bacterium-free co-culture of *Chlamydomonas* sp. and *Scenedesmus* sp. (A-S); red diamonds: bacterium-free co-culture of *Chlamydomonas* sp. and *Scenedesmus* sp. without sulfur deprivation (A + S).

H_2_ accumulated in the reactor headspace and concomitantly O_2_ disappeared in time when a mixture of *Scenedesmus* sp. and *Chlamydomonas* sp. was cultivated under nonsterile conditions together with their natural mutualistic bacterial partners (AB + S culture), which consumed the O_2_ produced by the algae. The results were compared with the H_2_ evolution by a mixture of the pure cultures of the two microalgae supplemented with hydrogenase-deficient *Escherichia coli* cells (AE + S culture) and by sulfur-deprived, bacterium-free algal cultures (A-S culture) (Figure 1). Striking differences were observed in terms of accumulated H_2_ yields and the commencement and duration of H_2_ evolution.

In the headspace of the growing algal-bacterial culture, the O_2_ level decreased from 21% to 4.5% in 12 h (Figure 1B). The low O_2_ level allowed H_2_ evolution by the algal biomass after 8 h and 1.15 ± 0.09 mL H_2_ L^−1^ was produced during the next 16 h, confirming earlier observations in similar systems (Figure 1A) [41].

The mutualistic bacteria were eliminated from the algal culture by photoautotrophic cultivation on minimal medium supplemented with rifampicin. H_2_ production was not observed of the bacterium-free algal culture (A + S), because O_2_ was not consumed by the mutualistic bacteria and the biosynthesis of the O_2_ sensitive hydrogenases was repressed (Figure 1A,B). The facultative anaerobic wild-type *E. coli* tends to consume O_2_ when it is available. Under anaerobic conditions, *E. coli* evolves H_2_ by using its own hydrogenases [42]. In order to eliminate the contribution of H_2_ production by *E. coli*, a pleiotropic hydrogenase mutant (Δ*hypF*) strain was used in these experiments so that only the facultative anaerobic property of this bacterium is functioning. Addition of *E. coli ΔhypF* cells and acetate to the pure algal culture (AE + S) efficiently reduced the level of O_2_ from 21% to 4% in 2 h. Pronounced H_2_ production accompanied this condition (1.52 ± 0.04 mL H_2_ L^−1^) (Figure 1A). The bacterial cell number in the spontaneously formed algal-bacterial culture (AB + S) was markedly lower than in the algal-*E. coli ΔhypF* co-culture (AE + S), which may explain why H_2_ generation by the AE + S started earlier than without the O_2_ scavenger *E. coli* strain (Figure 1). These data were compared with the H_2_ production by the mixture of the pure algal strains using the photoheterotrophic TRIS-acetate-phosphate medium (TAP) and employing the sulfur-deprivation method [43,44]. The sulfur-deprived pure *Scenedesmus* sp. and *Chlamydomonas* sp. mixture (A-S culture) became anaerobic after 20 h as opposed to the 2 to 8 h in the case of AB + S and AE + S. H_2_ evolution starts when anaerobic conditions are established; therefore, the difference in time required to reach anaerobicity is critical for the efficacy of the process. Additional benefits from practical aspect are the lower cost of alga production under nonsterile conditions and the elimination of labor- and cost-intensive transfer of algae into the sulfur-deficient medium.

The highest level of H_2_ generation by the A-S (1.91 ± 0.12 mL H_2_ L^−1^) was reached after 4 days (Figure 1A), which exceeded the H_2_ production of the AE + S culture only by about 20%. In view of the exceptionally thick cell walls of the *Scenedesmus* strains, the H_2_ productivity may have been partly diffusion-limited in the mixed algal culture, which may explain the lower H_2_ yield of A-S relative to the pure culture of sulfur-deprived *Chlamydomona*s sp. 549 strain (2.63 ± 0.04 mL H_2_ L^−1^) reported earlier [41]. Taken together, these experiments demonstrated that algal-bacterial natural mutualistic consortia are superior to the bacterium-free sulfur-deprived algal cultures from the aspect of H_2_ evolution.

There are two possible reasons why the H_2_ production ceased after about 24 h in the algal-bacterial co-cultures cultivated in TAP medium (see the ‘Materials and methods’ section). First, the H_2_ yield depends on the H_2_ partial pressure in a closed system [45]. Removal of the product H_2_ from the headspace allows the extension of the production time, leading to sustainable H_2_ evolution (data not shown). Secondly, in separate experiments, we have demonstrated that the depletion of acetate also results in a rapid loss of the mutualistic bacteria [41]. This can be remedied by the systematic addition of acetic acid to the system. Acetate is a low-value commodity produced in a number of anaerobic fermentative processes. The limiting factors of this bioH_2_ production methodology appear to be relatively easy to overcome. H_2_ production by algae under nonsterile conditions may make this approach economically viable on a large scale.

### Biogas production from algal-bacterial mixed biomass

**Figure 2:**
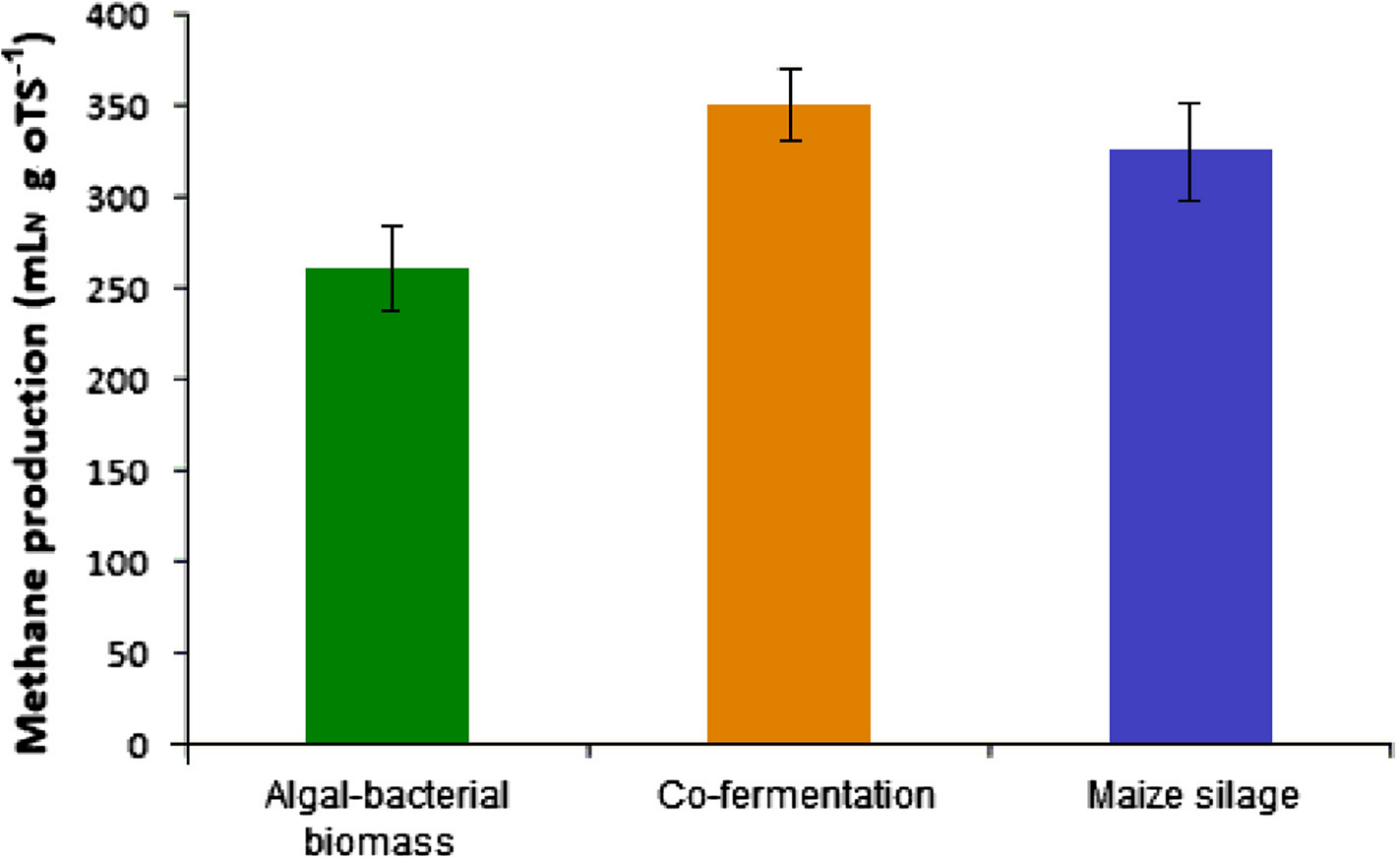
**Specific CH**_**4**_ **production from the various biomasses.**

The levels of biogas production from the various biomass substrates were determined after a 1-month of start-up and stabilization phase, that is, in weeks 1 to 4 of the experiment. During this time, the reactors were fed with the AB + S substrate to ensure that all the remaining and digestible biomass from the inoculum (containing pig slurry and maize silage) had been degraded and did not contribute to the biogas formation. Gas production data were collected during weeks 5 to 9, when the evolved gas was produced from the AB + S biomass. Biogas generation from the algal-bacterial mixture was compared with co-fermentaion of the alga-rich biomass and maize silage, and reactors fed with maize silage were used as controls. The CH_4_ concentration in the gas made from the AB + S biomass substrate was 58% to 61%, which is comparable to previous findings [25,26,46]. The biogas CH_4_ content from maize silage alone was 50% to 52%, as found previously [47]. The co-fermentation of algal-bacterial biomass with maize silage, in a ratio of 1:1, on the basis of organic dry matter (oDM), yielded a CH_4_ content of 54% to 57%, an intermediate value between those for maize silage and the algal-bacterial biomass. The daily average generated biogas volumes were as follows: from maize silage 3.20 L day^−1^, from co-fermentation 3.15 L day^−1^, and from algal-bacterial mixture 2.20 L day^−1^. Figure 2 shows the specific average CH_4_ production values (mL) calculated for g oDM^−1^.

For the appreciation of the potential value of the AB + S biomass as biogas substrate, its advantages relative to the widely used maize silage have to be taken into account. Most importantly, the AB + S biomass can be cultivated under nonsterile conditions on lands not useful for agricultural production and can be continuously harvested during extended period of the year. Although several technical issues related to the large-scale production of AB + S biomass for energetic purpose remain to be elaborated, this material may effectively replace a large portion of maize silage in the biogas reactors.

#### VOAs/TAC ratio indicated stable operation

**Figure 3:**
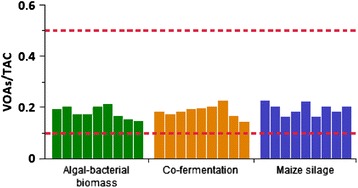
**Weekly measured VOAs/TAC ratios.** The area between the dashed red lines indicates the optimum range.

The ratio of the volatile organic acids (VOAs) and the total alkaline capacity (TAC) is an appropriate measure of the functional stability of the anaerobic digestion process [48,49]. A VOAs/TAC ratio below 0.1 means that the reactor needs feeding, whereas at a ratio ≥0.5 the biomass input is excessive and the process is out of balance. During the experiments, the average content of VOAs was 1.5 g L^−1^ and the average TAC was between 9 and 10 g CaCO_3_ L^−1^ in all cases. Figure 3 shows the weekly measured VOAs/TAC ratios.

A constant value of VOAs/TAC is a reliable indicator of a stable fermentation process. The organic loading rate was on the low side and allowed stable and balanced operation.

#### NH_4_^+^ accumulation

**Figure 4:**
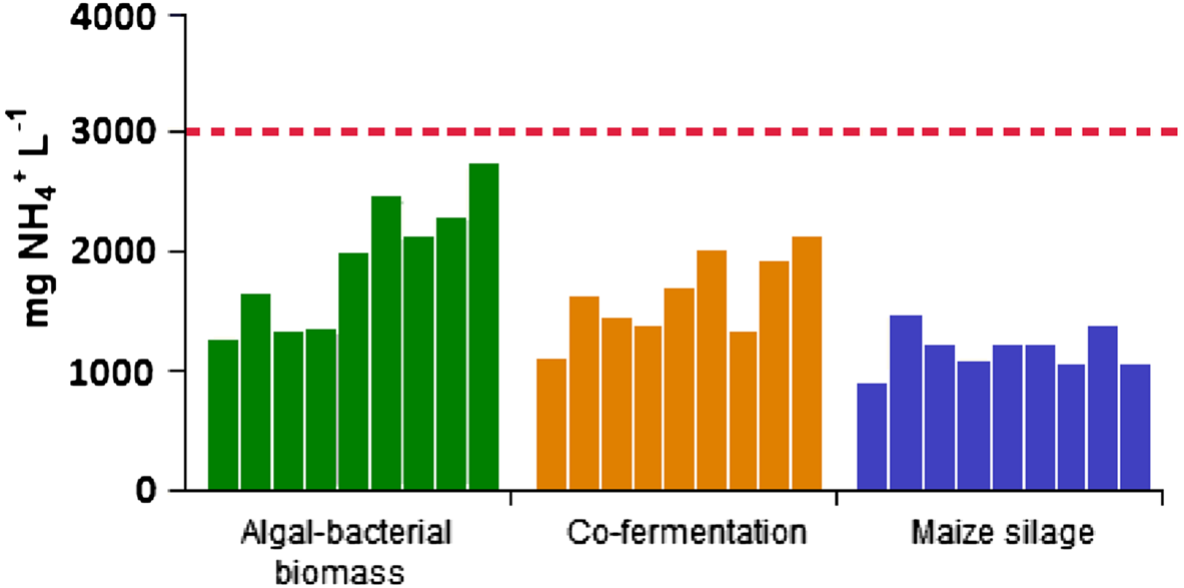
**Weekly measured NH**_**4**_^**+**^ **concentrations.** The dashed red line indicates the highest value recommended by the various studies.

From the decomposition of nitrogen-containing compounds, ammonia (NH_3_) is formed, which is present in the aqueous medium in the form of ammonium ion (NH_4_^+^) [50]. Values above 3,000 mg NH_4_^+^ L^−1^ may have a negative effect on the methanogenic community [51,52]. During the anaerobic fermentation, slight fluctuations in the weekly NH_4_^+^ concentrations were observed. In the case of using the algal-bacterial mixture, the NH_4_^+^ content tended to increase but remained under the critical 3,000 mg NH_4_^+^ L^−1^ level (Figure 4). Co-fermentation efficiently balanced this elevated NH_4_^+^ level.

#### The effect of the C/N ratio

**Figure 5:**
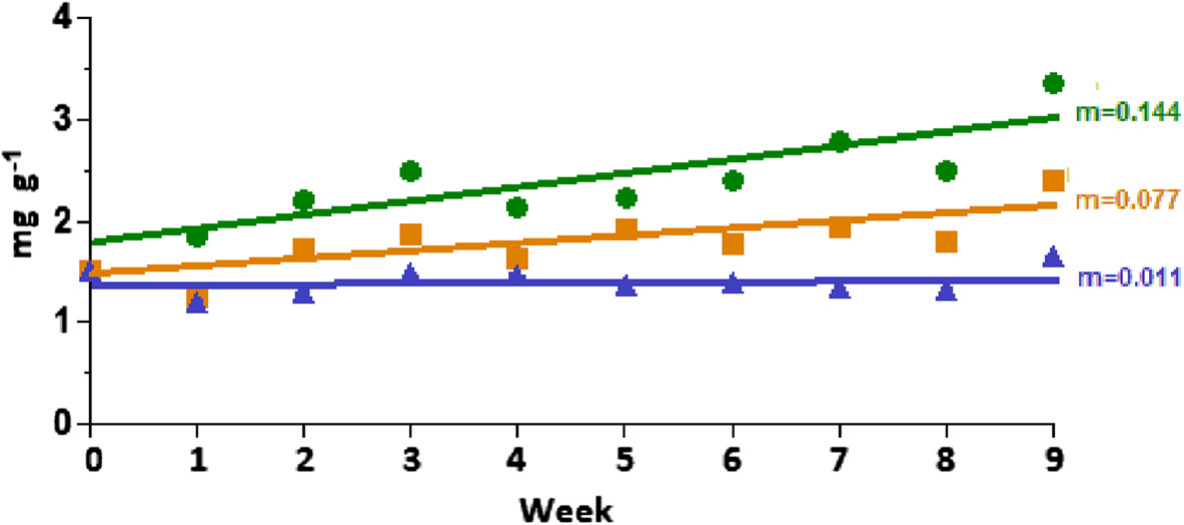
**Changes in N content during the AD of various substrates.** Green: AB + S, orange: co-fermentation, blue: maize silage.

The ideal C/N ratio for AD is 20 to 30 [53,54], because the microbes in the anaerobic reactor can utilize carbon (C) 20 to 30 times faster than nitrogen (N) [54]. The risk of C starvation increases if the C/N ratio is lower than 20; the methanogens are inhibited by the high NH_3_ accumulation, making the AD process vulnerable. At the other end of the spectrum, if the C/N ratio exceeds 30, the concentration of volatile fatty acids escalates, leading to process inhibition. The C/N ratios of the substrates used in this work are presented in Table 1. During the fed-batch continuous AD of microalgae and their mutualistic bacterial flora (AB + S), the nitrogen content increased. The initial C/N ratio of the AB + S biomass was low, 5.3. The nitrogen content increased as the fermentation progressed (Figure 5), accompanied by a slight but persistent free N concentration increase. Co-fermentation of the algal-bacterial biomass with maize silage, which had a C/N ratio of 45.3, led to a less pronounced N accumulation, indicating a buffering effect of the maize silage. In the reactors fed with maize silage alone, the N level remained nearly constant (Figure 5).

**Table 1:** **The initial substrate compositions**

**Substrate**	**Wet mass N (mg g** ^**−1**^ **)**	**Wet mass C (mg g** ^**−1**^ **)**	**C/N ratio**	**TS (%)**	**oDM (%)**
Maize silage	4.35	196.86	45.3:1	41.19	94.59
Algal-bacterial mix	18.65	98.33	5.3:1	30.30	97.71

TS = total solids, oDM = organic dry material.

Olsson *et al*. reported that feeding AD reactors with a high proportion of microalgal biomass in co-fermentation with waste water sludge had a negative effect under both thermophilic (55°C) and mesophilic (37°C) conditions, possibly because of the high N content of the microalgal biomass [55]. Co-fermentation of a microalgal biomass with waste paper improved the AD performance [56], presumably in consequence of the higher C/N ratio of the mixed substrate and the induction of cellulase biosynthesis by the paper sludge. In our case, co-fermentation of the algal-bacterial biomass with the cellulose-rich maize silage likewise enhanced the biogas productivity.

#### Microbial community

The composition of the microbial community was established at four time points: at the start of feeding with the selected substrate (start), 1 week later (week 1), when the system was working at full capacity (week 5), and at the end of the process (week 9). The microbial community compositions of the substrates were determined separately.

### Microbiological compositions of the substrates

**Figure 6:**
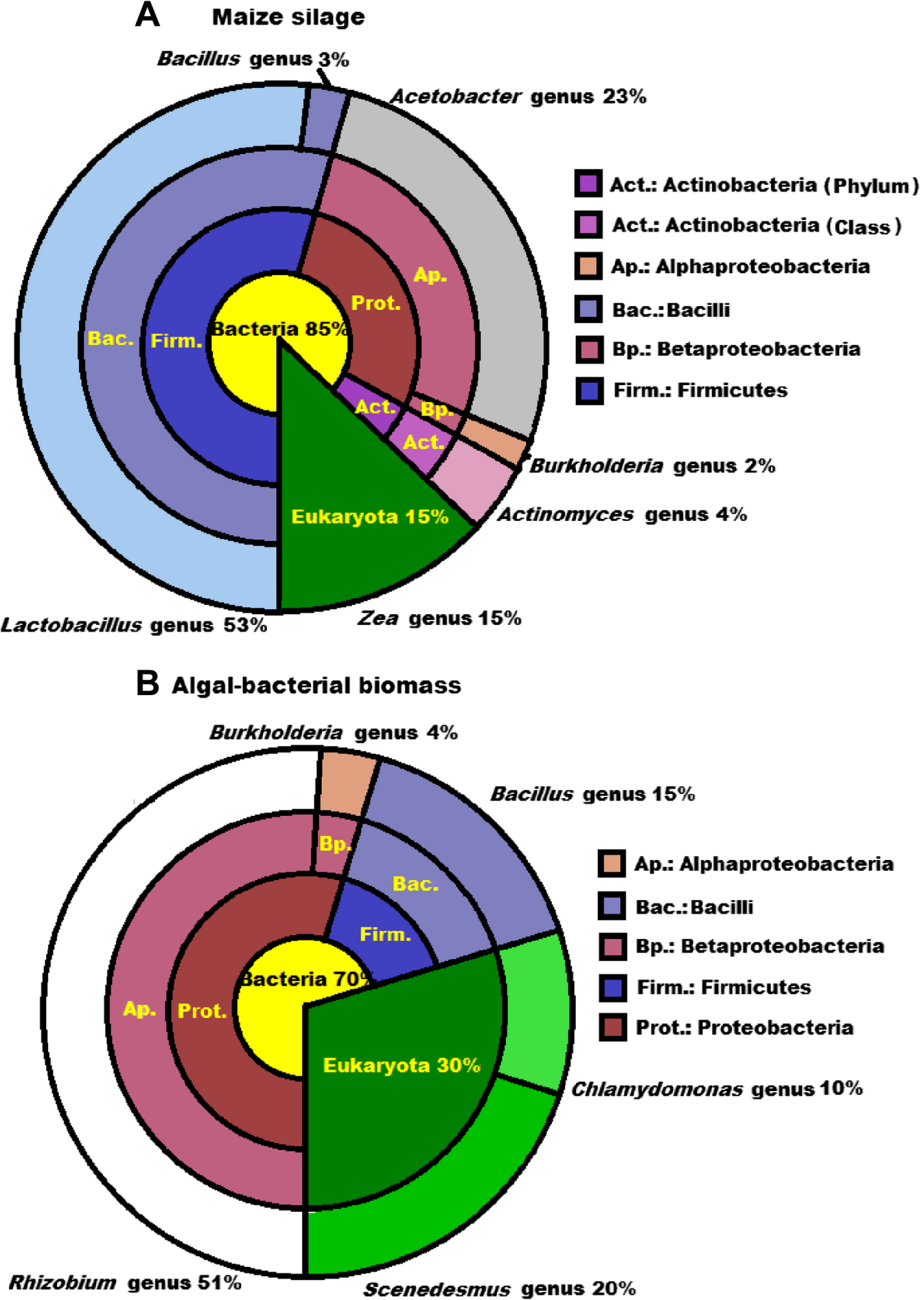
**Microbial compositions of the substrates: (A) Maize silage, (B) AB + S.** The communities at domain, phylum, class, and genus levels are indicated.

The microbial flora of the maize silage included representatives of the genera *Lactobacillus* and *Acetobacter*, as expected (Figure 6A). Lactobacilli produce lactate from mono- and disaccharides [57]. Upon ensilaging, the accumulating acid decreases the pH and preserves the green plant material. Members of the genus *Acetobacter* primarily contribute to acetate production [58].

The mixture of *Chlamydomonas sp*. and *Scenedesmus sp*. microalgae was cultivated under nonsterile conditions and contained copious amounts of the mutualistic bacteria (Figure 6B). *Rhizobium* species predominated in the bacterial population. *Rhizobium* is well known for its syntrophic interaction with plants and mutualism has also been observed in the cases of several microalgal species [36,39]. The major probable driving force behind this association is vitamin B_12_, which the algae needs for growth but cannot synthesize. *Rhizobium* is there to supply the algae with vitamin B_12_ in exchange for fixed carbon. The growth rate and the resistance to environmental stresses improve as a result of the algal-bacterial interactions [36,39]. Other forms of mutualism between microalgae and bacteria have also been recognized [31-34].

### The biogas-producing microbial community

The distribution of the microbial taxa in the biogas-producing microbial community at the beginning of the experiments was very similar to that found in earlier studies on reactors fed with pig manure and maize silage [59], in good agreement with starting the reactors with inocula from a mesophilic industrial biogas facility digesting such substrates. These results may therefore be regarded as an internal control validating the metagenome sequencing method. In the following detailed analysis of the metagenomic results, the unidentified sequences are disregarded.

#### Microbial community of maize silage AD (domain Bacteria)

**Figure 7:**
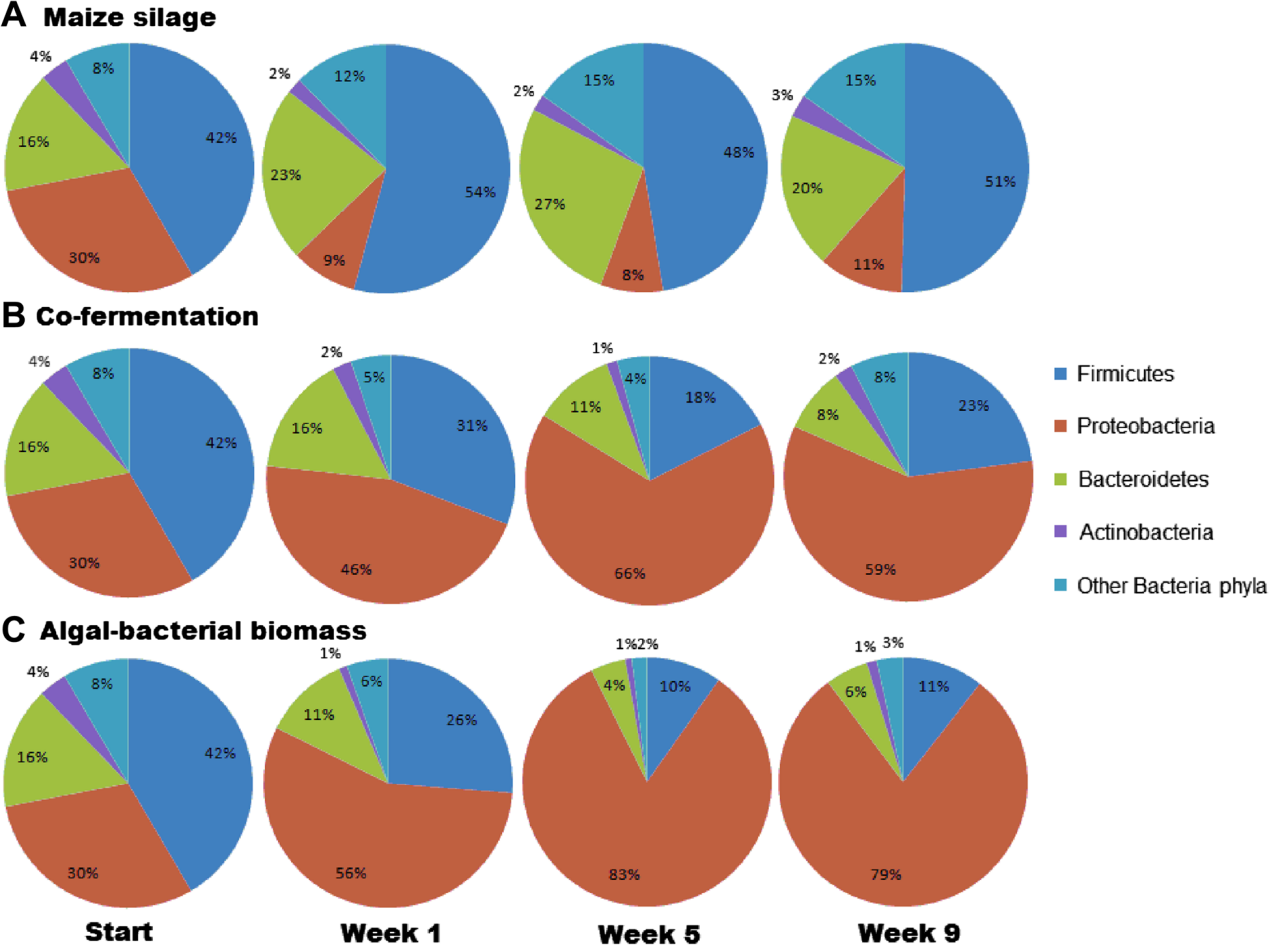
**Changes in the domain Bacteria of the microbial community at phylum level. (A)** Maize silage, **(B)** co-fermentation, and **(C)** algal-bacterial biomass.

**Figure 8:**
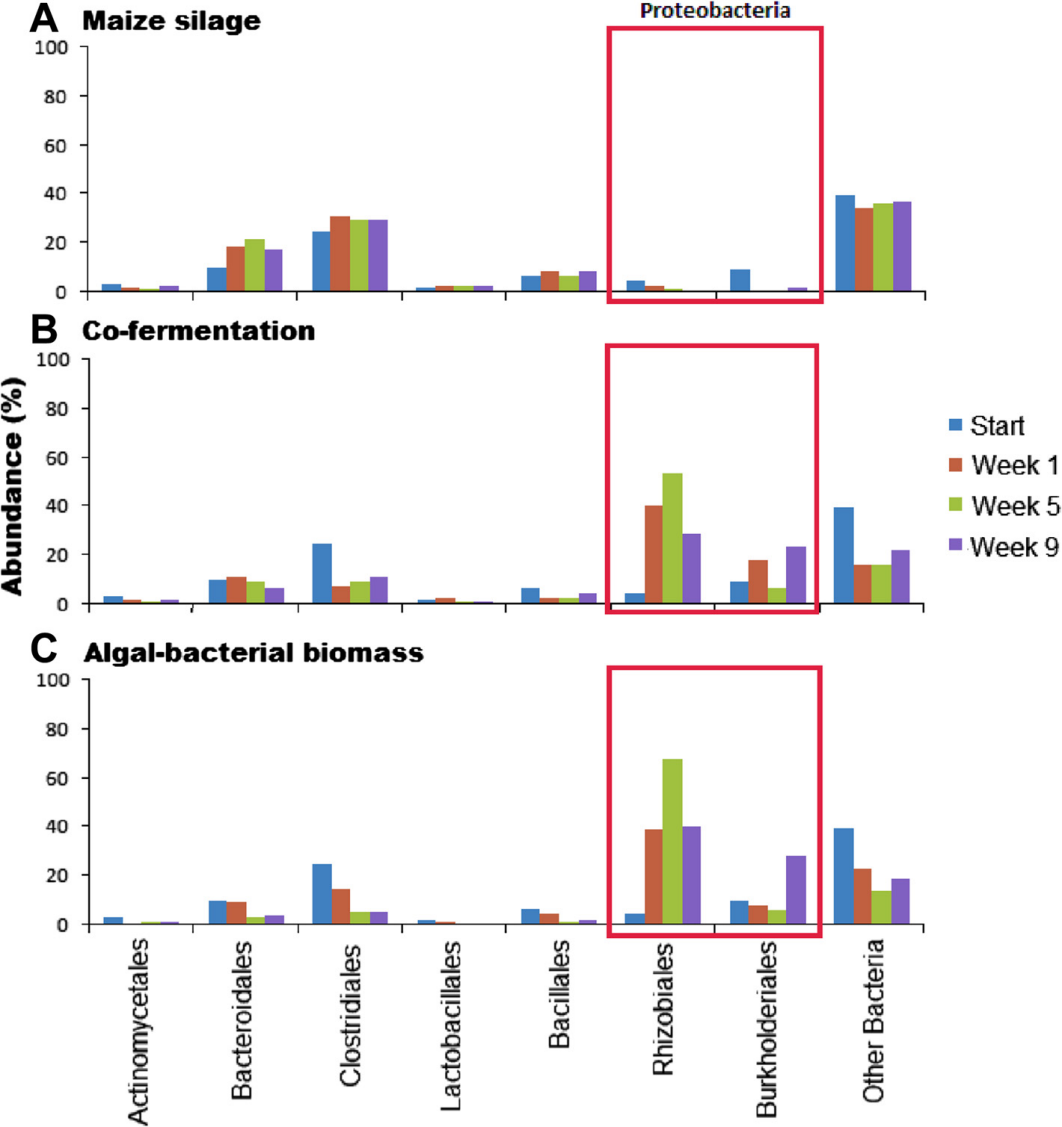
**Changes in the domain Bacteria of the microbial community at the order level. (A)** Maize silage, **(B)** co-fermentation, and **(C)** algal-bacterial biomass.

Only relatively minor and trivial rearrangements occurred in the relative distribution of the bacterial taxa during the experimental period (Figure 7). This is not surprising in view of the fact that the reactors were sustained on pig manure and maize silage prior to the start of the experiment. In the domain *Bacteria*, the most abundant strains belong in the phylum *Firmicutes*. Pronounced changes were seen in the phylum *Proteobacteria*. Some of the *Proteobacteria* were apparently displaced by *Firmicutes* and *Bacteroidetes*. In the phylum *Firmicutes*, the orders *Clostridiales* and *Bacteroidales* predominated (Figure 8). Among the *Clostridiales*, the genus *Clostridium* increased in abundance, followed by the genus *Bacillus*. In the order *Bacteroidales*, the genus *Bacteroides* predominated (data not shown).

#### Microbial community of co-fermentation (domain Bacteria)

Co-fermentation of the algal-bacterial mixture with maize silage provoked major changes in the composition of the bacterial community within a week as compared with the AD of maize silage (Figures 7 and 8). At the starting time point, there was no difference between the reactors fed with the various substrate compositions, indicating that the same microbial community was established during the start-up phase and the initial conditions were therefore identical. Supplying the reactors with a 1:1 mixture of microbial biomass and maize silage instigated a rearrangement within the biogas-producing microbial community. Representatives of the phylum *Proteobacteria* gradually predominated in the community, and within the taxon, the orders *Rhizobiales* and *Burkholderiales* prevailed (Figure 8). At higher resolution, a marked accumulation of the genera *Rhizobium* and *Burkholderia* was evident as the experiment progressed, although the phylum *Proteobacteria* displayed a diverse representation at the start. At the same time, members of the phylum *Firmicutes* and to a lesser degree those belonging to the phylum *Bacteroidetes* lost their significance within the AD community. The majority of bacteria belonging in these taxa have gained a reputation as outstanding cellulose degraders and H_2_ producers, both of these metabolic activities being crucial for efficient biogas production from plant biomass.

#### Microbial community of microalgal-bacterial fermentation (domain Bacteria)

**Figure 9:**
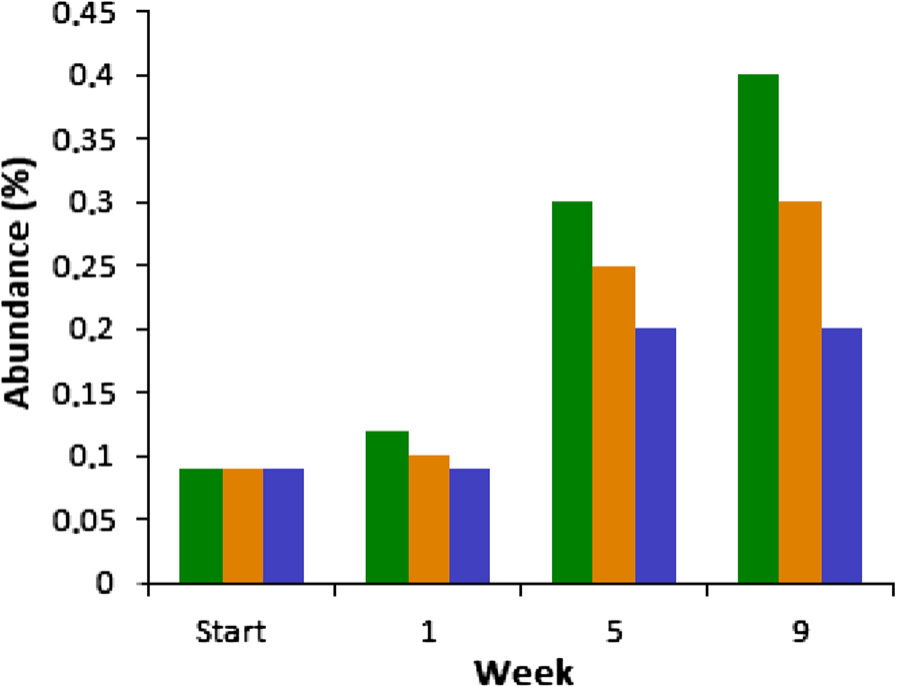
**Eukaryotic sequences in the reactors.** Green: AB + S, orange: co-fermentation, blue: maize silage.

A noteworthy fast response by the biogas-producing microbial community was observed when the substrate added to the reactors was changed from the mixture of pig slurry and maize silage to the algal-bacterial biomass. The reaction was less pronounced, but similar when the reactors were fed with a 1:1 mix of plant and microbial biomasses, as discussed above. The main outcome of this reorganization was the predominance of the phylum *Proteobacteria*, which surpassed the phyla *Firmicutes* and *Bacteroidetes*. The genera *Rhizobium* and *Burkholderia* were introduced into the reactor with the substrate (Figure 6) and accumulated in time (Figure 8), in spite of the relatively low daily organic loading rate. Either the decomposition was too slow to convert the total administered bacterial biomass to biogas, or the *Rhizobia* multiplied faster than their anaerobic degradation. *Rhizobium* species survive in free living form under anaerobic conditions, taking advantage of their nitrate respiration capability [60,61], but it is unlikely that their growth rate exceeds that of the anaerobic degradation by the biogas microbial community. The substrate was stored at −20°C for about 3 months before being fed into the reactors. It seems likely that the build-up of *Proteobacteria* in time is due to their relatively slow decomposition under the AD conditions. In this respect, it is noteworthy that the relative abundance of eukaryotic sequences in the reactors also increased in time (Figure 9). The eukaryotic DNA accumulation from the algal biomass was twice that from the maize, suggesting that the algal cell wall may be more resistant than that of the maize silage to microbial degradation.

This implies that the biogas potential of the algal biomass is higher than that of the bacterial biomass, although a correct mass balance is difficult to achieve because of the complexity of the organic materials in the reactor.

### The domain Archaea

**Figure 10:**
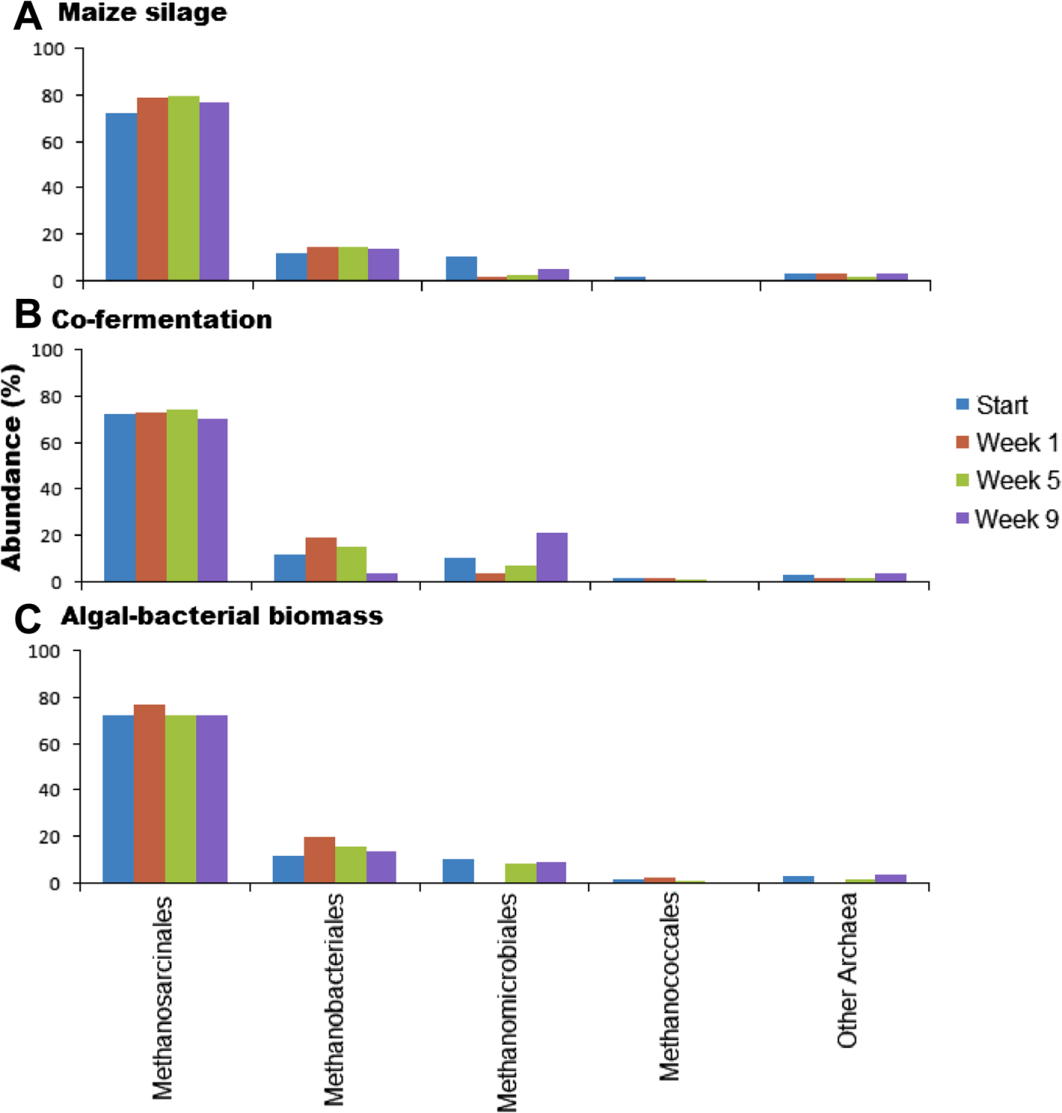
**Distribution of the domain Archaea in the microbial community at the order level. (A)** Maize silage, **(B)** co-fermentation, and **(C)** algal-bacterial biomass.

In the domain *Archaea*, a microbial composition was found that was distinct from those observed in previous studies in reactors fed with ‘conventional’ substrates [59,62-66]. The class *Methanomicrobia* represented the domain *Archaea* in great abundance. The *Methanomicrobia* are able to operate all three routes of methanogenesis [67]. The order *Methanomicrobiales* was the most prevalent from the start, and at higher resolution, the members of the genus *Methanosarcina* predominated. Seasonal changes or other uncontrolled factors may also be responsible for these alterations in the AD communities [68-70]. At any rate, the genus *Methanosarcina* remained predominant in all fermentations tested in this study (Figure 10). Interestingly, in a previous study, involving the use of next-generation sequencing *mcrA* genes, the order *Methanosarcinales* was also found to be predominant in the AD of a mixture of waste water sludge and a nonsterile, unidentified algal biomass [27]. In the Archaeal community converting that substrate to biogas, the acetotrophic genus *Methanosaeta* (order *Methanosarcinales*) was identified as the prevailing taxonomic unit. Members of the genus *Methanosaeta* were present in our study too, although in less abundance.

## Conclusions

A combination of bioH_2_ and biogas production by a mixture of nonsterile microalgae and natural bacterial flora was demonstrated. In a closed system, the mutualistic bacteria consumed the O_2_ evolved by the algae and created a sufficiently anaerobic environment for algal H_2_ evolution without damaging the photosynthetic apparatus of the algae. With the help of the bacterial partners, the algae succeeded in capturing light energy by photosynthetic water splitting and evolved H_2_ at the same time without the need for further manipulation of the system, such as sulfur deprivation.

H_2_ production through the use of a mixture of microalgae and syntrophic bacteria started earlier than the H_2_ evolution following sulfur deprivation, although sulfur-deprived *C. reinhardtii* produced bioH_2_ for a longer period of time.

AD and biogas evolution from the nonsterile microalgal-bacterial biomass yielded a gas enriched in CH_4_ relative to the commonly used maize silage. The specific biogas production estimated on the basis of the organic material input, however, was smaller than that from maize silage. The addition of maize silage to the algal-bacterial mixed biomass increased the C/N ratio considerably and improved the balanced digestibility of the microbial biomass.

The metagenome analysis of the microbial communities present in the AD reactors revealed the persisting impact of the microalgae and their bacterial companions on the composition of the AD microbial community within a few days. The large amount of bacteria belonging in the genera *Rhizobium* and *Burkholderia*, dosed together with the microalgal biomass, significantly changed the bacterial community composition. Co-fermentation of the algal-bacterial biomass with maize silage compensated somewhat for the *Rhizobium* and *Burkholderia* predominance due to the 50% lower loading of the microbial biomass on an organic dry matter basis. In the control reactors fed with maize silage, the microbial taxa belonging in the phyla *Firmicutes* and *Bacteroidetes* persisted.

Interestingly, the pronounced alterations observed in the domain *Bacteria* did not affect the composition of the domain *Archaea*. The order *Methanosarcinales* predominated in the Archaeal community regardless of the substrate composition.

## Materials and methods

### Cultivation of pure and mixed cultures

The *Chlamydomonas* sp. and *Scenedesmus* sp. algae and their mutualistic bacteria (AB + S culture) were obtained as algal strain 810 from the Mosonmagyaróvár Algal Culture Collection (MACC) of Hungary. The purified algal mixture was maintained and cultivated on TP (TRIS-phosphate) medium supplemented with rifampicin. The TP medium is a modified TAP (TRIS-Acetate-Phosphate) medium where acetate is replaced with HCl. The TAP and TP plates were incubated under 50 μmol m^−2^ s^−1^ light intensity at 25°C. Algae used for H_2_-evolution experiments were harvested as fresh cultures grown on TP-agar plates supplemented with rifampicin and transferred into liquid TAP medium [41,71,72]. The algal stock solutions were equally distributed into 40-mL Hypo-Vial bottles, resulting in a final volume of 35 mL and a final optical density (OD_750_) of 0.7. *E. coli ΔhypF,* a hydrogenase-deficient strain, was grown on LB (Luria-Bertani medium) plates at 30°C in the dark.

The original algal-bacterial culture, used for H_2_ production experiments, was pre-grown in TAP medium. The medium of the pre-grown stock culture was changed to fresh TAP medium by centrifugation. It was diluted to OD_750_ of 1.2. Bottles were sealed with butyl rubber stoppers and aluminum caps. All experiments were performed in at least three parallel repetitions.

### Algal-bacterial culture scaling up for biomass production

For biomass production, an unpurified culture of a *Scenedesmus* sp. and *Chlamydomonas* sp. mixture, as obtained from the MACC collection, was cultivated in 13-L polycarbonate vessels under 50 μmol m^−2^ s^−1^ light intensity at 25°C for 5 days before harvesting. The biomass yield was approximately 2 g L^−1^. The microbial biomass was harvested by using a cross-flow centrifuge and the harvested biomass was stored at −20°C until utilization.

### Gas chromatographic analyses

The H_2_ and O_2_ levels in the headspace of the Hypo-Vial bottles were measured by gas chromatography. An Agilent 7890A gas chromatograph (Agilent Technologies, Santa Clara, USA), equipped with a thermal conductivity detector and an Agilent HP-Molsieve column (length 30 m, diameter 0.320 mm, film 12.0 μm; Agilent Technologies, Santa Clara, USA) was used in splitless mode. Linde HQ argon 5.0 (Linde Group, Munich, Germany) was used as carrier and reference gas. The temperatures of the injector, TCD detector, and column were kept at 150°C, 160°C, and 60°C, respectively. The column pressure was 47.618 psi. The flow rate of the column was 12 mL min^−1^. Samples (50 μL) were analyzed. Three biological replicates were used for the measurements. A H_2_ calibration curve was used to determine accurate gas volumes. Serial dilutions of pure H_2_ gas were prepared in 25-mL gas-tight vials, and identical volumes were injected into the gas chromatograph: data from three replicates were used to draw the H_2_ calibration curve.

### Anaerobic fermentation and biogas analysis

Anaerobic fermentations were carried out in 5-L continuously stirred tank reactors [73], and the fed-batch operational mode was used. The reactors were operated by using a pig manure and maize silage mixture [59] until the biogas production stabilized. This start-up period lasted for 4 to 5 weeks. Feeding with the various substrate compositions was started thereafter. One of the reactors was fed with a AB + S loading of 1 g oDM L^−1^ day^−1^, an identical reactor was supplied with AB + S and corn silage (0.5 + 0.5 g oDM L^−1^ day^−1^), and the control received only corn silage (1 g oDM L^−1^ day^−1^). The initial parameters of the substrates are summarized in Table 1. Heating was maintained by means of an electronically heated jacket which surrounded the cylindrical apparatus. Temperature was measured with a bimetallic sensor and was maintained constant at 37°C ± 1.0°C. The pH was between 7 and 8, and the redox potential was < −500 mV. The generated gas and its quality were measured daily after the 1-month start-up (weeks 1 to 4) and stabilization phase on the designated substrate. Gas volumes were measured with thermal mass flow devices (DMFC; Brooks Instrument, Hatfield, USA) attached to each gas exit port. The composition of the evolved biogas was measured with a gas chromatograph (6890 N Network GC System, Agilent Technologies, Santa Clara, USA) equipped with a 5 Å molecular sieve column (length 30 m, I.D. 0.53 megabore, film 25 μm). Ultrapure N_2_ was used as carrier gas.

### Determination of fermentation parameters

#### oDM

The dry organic matter content was quantified by drying the biomass at 105°C overnight and weighing the residue giving the dry mass content. Further heating of this residue at 550°C provided the organic dry mass content.

#### Density measurement

Sample density was measured with a MINIDENS automatic density meter (Grabner Instruments, Wien, Austria).

#### C/N

To determine C/N, an Elementar Analyzer Vario MAX CN (Elementar Group, Hanau, Germany) was employed. The equipment works using the principle of catalytic tube combustion under an O_2_ supply at high temperatures (combustion temperature: 900°C, postcombustion temperature: 900°C, reduction temperature: 830°C, column temperature: 250°C). The desired components were separated from each other with the aid of specific adsorption columns (containing Sicapent (Merck, Billerica, USA), in C/N mode) and determined in succession with a thermal conductivity detector. Helium served as flushing and carrier gas.

#### NH_4_^+^-N

For the determination of NH_4_^+^ ion content, the Merck Spectroquant Ammonium test (1.00683.0001) (Merck, Billerica, USA) was used. At the beginning of the experiment the NH_4_^+^ − N was 1,100 mg L^−1^.

#### VOAs/TAC

Five grams of fermenter sample was taken for the analysis and diluted to 20 g with distilled water. The subsequent process was carried out with Pronova FOS/TAC 2000 Version 812-09.2008 automatic titrator (Pronova, Berlin, Germany). At the beginning of the experiment the VOAs/TAC ratio was 0.2.

### DNA isolation for metagenomic studies

Two-milliliter samples, taken from the reactors, were used for total community DNA isolation. The extractions were carried out with a slightly modified version of the Zymo Research kit (D6010, Zymo Research, Irvine, USA). Parallel samples from each reactor were lysed with three different lysis mixes (Table 2). After lysation (bead beating), the Zymo Research kit protocol was followed. The quantity of DNA was determined in a NanoDrop ND-1000 spectrophotometer (NanoDrop Technologies, Wilmington, USA) and a Qubit 2.0 Fluorometer (Life Technologies, Carlsbad, USA). DNA purity was tested by agarose gel electrophoresis and with an Agilent 2200 Tape Station (Agilent Technologies, Santa Clara, USA).

**Table 2:** **Lysis conditions for total community DNA preparation**

	**Lysozyme** ^**a**^ **(μL)**	**10% CTAB** ^**b**^ **(μL)**	**Genomic CTAB lysis buffer** ^**c**^ **(μL)**	**Qiagen buffer** ^**d**^ **(μL)**	**Zymo buffer** ^**e**^ **(μL)**
A	-	100	-	100	550
B	250	100	-	100	300
C	250	-	300	200	-

^a^100 mg mL^−1^ (Applichem, Barcelone, Spain). ^b^Cetyltrimethylammonium bromide (*w*/*v*). ^c^1 M Tris-HCl 100 mL, 500 mM EDTA 50 mL, 5 M NaCl 300 mL, 10% CTAB, 20% SDS, pH = 8 (Wirth *et al*. [64]). ^d^ASL buffer from Qiagen QIAamp DNA Stool miniprep kit (51504, Qiagen, Limburg,Netherlands). ^e^From Zymo Research Fecal DNA kit (Zymo Research, D6010).

### Next-generation DNA sequencing and data handling

The sample preparation for total metagenome sequencing of the pooled samples was carried out following the recommendations of the Ion Torrent PGM sequencing platform (Life Technologies, Carlsbad, USA). Sequencing was performed with Ion Torrent PGM 316 chips. The reads were analyzed and quality values were determined for each nucleotide. The 150 to 250 nucleotide-long individual sequences were further analyzed by using the MG-RAST software package [74], which is a modified version of Rapid Annotations based on Subsystem Technology (RAST). The MG-RAST server computes results against several reference datasets (protein and ribosomal databases) [75]. The generated matches to external databases were used to compute the derived data [59,76].

## Abbreviations

A + S: mixture of *Chlamydomonas* sp., and *Scenedesmus* sp., non-sulfur deprived
AB + S: mixture of *Chlamydomonas* sp., *Scenedesmus* sp., and mutualistic bacteria, non-sulfur deprived
AD: Anaerobic digestion
AE + S: mixture of *Chlamydomonas* sp., *Scenedesmus* sp., and *Escherichia coli* Δ*hypF*, non-sulfur deprived
A-S: mixture of *Chlamydomonas* sp., and *Scenedesmus* sp., sulfur deprived
oDM: organic dry matter
TAC: Total alkaline capacity
TAP: TRIS-acetate-phosphate medium
TP: TRIS-phosphate medium
VOS: Volatile organic acids
